# Antidepressants Usage and Risk of Pneumonia Among Elderly Patients With the Parkinson's Disease: A Population-Based Case-Control Study

**DOI:** 10.3389/fmed.2022.740182

**Published:** 2022-02-18

**Authors:** Wei-Yin Kuo, Kuang-Hua Huang, Yu-Hsiang Kuan, Yu-Chia Chang, Tung-Han Tsai, Chien-Ying Lee

**Affiliations:** ^1^Department of Health Services Administration, China Medical University, Taichung, Taiwan; ^2^Department of Pharmacology, Chung Shan Medical University, Taichung, Taiwan; ^3^Department of Pharmacy, Chung Shan Medical University Hospital, Taichung, Taiwan; ^4^Department of Long Term Care, National Quemoy University, Kinmen, Taiwan; ^5^Department of Healthcare Administration, Asia University, Taichung, Taiwan; ^6^Department of Medical Research, China Medical University Hospital, Taichung, Taiwan

**Keywords:** antidepressants, pneumonia, the elderly, Parkison's disease, pharmacoepidemiology

## Abstract

The patients with Parkinson's disease (PD) are associated with a higher risk of pneumonia. Antidepressants exert an anticholinergic effect in varying degrees and various classes of antidepressants also can produce a different effect on immune function. The relationship between the risk of pneumonia and the use of antidepressants among elderly patients with PD is unknown. The study investigated the risk of pneumonia associated with the use of antidepressants in elderly patients with PD. This case-control study was based on data from the longitudinal health insurance database in Taiwan. We analyzed the data of 551,975 elderly patients with PD between 2002 and 2018. To reduce the potential confounding caused by unbalanced covariates in non-experimental settings, we used propensity score matching to include older patients without pneumonia to serve as the comparison. The antidepressants in the study included tricyclic antidepressants (TCAs), monoamine oxidase inhibitors (MAOIs), selective serotonin reuptake inhibitors (SSRIs), serotonin, and norepinephrine reuptake inhibitors (SNRIs). The conditional logistic regression was used to investigate the association between antidepressants and pneumonia. Control variables in the study included sex, age, income level, urbanization, Charlson comorbidity index score, and comorbidities related to pneumonia. In terms of TCAs users, compared with patients not receiving TCAs, current users had a lower risk of incident pneumonia (adjusted odds ratio [*aOR*] = 0.86, 95% *CI* = 0.82–0.90) and recent users (a*OR* = 0.83, 95% *CI* = 0.80–0.87). In terms of MAOIs users, current users had a lower risk of incident pneumonia (a*OR* = 0.88, 95% *CI* = 0.83–0.93), recent users (a*OR* = 0.89, 95% *CI* = 0.85–0.93). In terms of SSRIs users, current users had a higher risk of incident pneumonia (a *OR* = 1.13, 95% *CI* = 1.01–1.17), recent users (a*OR* = 1.01, 95% *CI* = 1.06–1.13), and past users (a*OR* = 1.19, 95% *CI* = 1.17–1.21). In terms of SNRIs users, past users had a higher risk of incident pneumonia (a*OR* = 1.07, 95% *CI* = 1.03–1.10). The incident pneumonia is associated with the use of individuals of different classes of antidepressants. The use of TCAs (such as, amitriptyline and imipramine) had a lower odds of incident pneumonia. The use of MAOIs (such as, selegiline and rasagiline) had a lower odds of pneumonia during recent use. The use of SSRIs (such as, fluoxetine, sertraline, escitalopram, paroxetine, and citalopram) and SNRIs (such as, milnacipran, and venlafaxine) had a higher odds of incident pneumonia.

## Introduction

Patients with neurodegenerative diseases such as Parkinson's disease (PD) commonly experience motor disturbance because of the degeneration of dopaminergic neurons (1). Neuro-inflammation is a harmful process and associated with the chronic neurodegenerative diseases such as PD (2). Cytokines are physiologically expressed in cells in the central nervous system and are particularly crucial during neural development, starting from the induction of the neuroepithelium (3). Oropharyngeal dysphagia is a common and clinically relevant symptom in patients with PD and may occur at any stage in the disease course. The majority of patients with early stage PD developed pharyngeal and esophageal impairment even before the clinical manifestation of dysphagia (4).

Increasing evidence suggests a relationship between PD and depression (5–8). A bidirectional relationship may exist between depression and PD (5). Inflammatory responses play a crucial role in the pathophysiology of depression (6). Patients with PD who develop depression receive antidepressants. However, antidepressants not only suppress the release of pro-inflammatory cytokines, but also stimulate the release of anti-inflammatory cytokines (7). A previous study reviewed the literature on the role of pro-inflammatory cytokines in depression to explore the immunomodulatory effects of antidepressants on patients with PD (8). A previous study conducted in Germany calculated the anticholinergic burden for drugs and reported that tricyclic antidepressants (TCAs) exert strong anticholinergic effects, whereas selective serotonin reuptake inhibitors (SSRIs) and serotonin and norepinephrine reuptake inhibitors (SNRIs) exert weak anticholinergic effects (9). In addition, a study conducted in Taiwan reported that older patients receiving anticholinergic medications have an increased risk of pneumonia (10). The anticholinergic actions of antidepressants may be associated with the increased risk of pneumonia (11).

However, different types of antidepressants may exert different anticholinergic effects; they can exert different effects on immune function. However, the association between various antidepressants and the incidence of pneumonia in older patients with PD remains unknown. To understand this association, whether the incident pneumonia is associated with the use of different classes of antidepressants in older patients with PD should be examined. Therefore, the present study investigated whether the incidence of pneumonia is associated with the use of various types of antidepressants in older patients with PD.

## Materials and Methods

### Database

This study is the secondary data analysis based on the longitudinal health insurance database (LHID) from 2001 to 2018 released by the Health and Welfare Data Science Center, Ministry of Health and Welfare (HWDC, MOHW). In this study, the LHID was from the Taiwan National Health Insurance (NHI) program that has enrolled up to 99% of citizens. Hence, the database is a nationally representative health database for Taiwan. The information in LHID, such as detailed clinical records of the outpatient department and hospitalization, diagnostic codes, and prescribing information, is high concordance between NHI claims records and self-reports of patients. Therefore, the LHID is frequently used for analyzing drug safety data, such as drug-induced pneumonia. The database is de-identified and HWDC provides scrambled random identification numbers for insured patients to protect the privacy of beneficiaries. The requirement for informed consent was waived. This study protocol was approved as a completely ethical review by the Central Regional Research Ethics Committee (CRREC) of the China Medical University, Taiwan (No. CRREC-109-011).

### Study Subjects

The study population comprised elderly patients (aged ≥ 65 years old) with PD (International Classification of Diseases, Ninth Revision, Clinical Modification [ICD-9-CM]: 332, ICD-10-CM: G20) from 2002 to 2018. The elderly patients who had principal diagnosed with pneumonia (ICD-9-CM: 480-486, ICD-10-CM: J12-J18) were the case group. The comparison was the elderly patients without any pneumonia diagnosis in the same year. To reduce the potential confounding caused by unbalanced covariates in non-experimental settings, we used propensity score matching (PSM) to match the elderly patients without pneumonia as the comparison in 1:4 ratio (12). The PSM is a statistical matching technique that is available to reduce potential confounding caused by unbalanced covariates in non-experimental settings. The propensity score is the probability calculated *via* the logistic regression model. In this study, the propensity score is the probability of the risk of incident pneumonia calculated *via* the logistic regression model. That score is a unit with certain characteristics that will be assigned to the case group and the comparison. The scores could be used to reduce or eliminate selection bias in observational studies by the characteristics of pneumonia patients and comparison. The characteristics we selected for matching were sex, age, income level, urbanization, and Charlson comorbidity index (CCI). The CCI was a measurement tool developed by Charlson et al. (13) that was consisted of 17 comorbidities and used widely to measure the burden of disease or case-mix with administrative data (14, 15). Some respiratory disease studies based on secondary data analysis were as well (10, 16–18). Some respiratory disease related to incident pneumonia was not contained in matching variables, such as asthma, chronic obstructive pulmonary disease (COPD). Due to chronic pulmonary disease (ICD-9-CM: 490-505, 506.4; ICD-10-CM: I27.8-9, J40–47, J60–67, J68.4, J70.1, J70.3) was contained in the CCI. In addition, some pneumonia-related diseases were not contained to the CCI. In reference to the previous study (10), the study used CCI for matching variables and used individual comorbidities for adjusting the risk of antidepressants and the incident pneumonia.

### Study Design

This study was a case-control study to investigate the risk of incident pneumonia associated with antidepressants among elderly patients with PD. We employed this appropriate study design primarily because pneumonia events were relatively rare, which were not suitable for implementing a cohort study design. After matching, we assigned an index date to the comparison as the same with the incident pneumonia date of the corresponding case-patients for 1-year observation. Approximately 1 year before, pneumonia was the observation period for each patient to access the antidepressants use. Exposure to antidepressants was classified as current, recent, and past, respectively. The definition of “current” was when the most recent prescription was within 30 days before pneumonia. Prescriptions within 31–90 days before the pneumonia were treated as “recent” exposures, while prescriptions of 90 days or more before the pneumonia were treated as “past” exposure. In addition, patients who had never been prescribed antidepressants before the pneumonia were the reference group. We defined the use of antidepressants by the following Anatomic Therapeutic Chemical classification (ATC) system codes. The ATC codes of antidepressants were all detailed as Appendix Table 1. The antidepressants of the study contained TCAs (Amitriptyline, Clomipramine, Doxepin, and Imipramine), Monoamine oxidase inhibitors (MAOIs) (Isocarboxazid, Selegiline, Rasagiline, Tranylcypromine, and Moclobemide), SSRIs (Paroxetine, Fluoxetine, Citalopram, and Fluvoxamine, Sertraline, Escitalopram), SNRIs (Duloxetine, Milnacipran, and Venlafaxine), and other antidepressants (Trazodone and Mirtazapine). Control variables in the study contained sex, age, income level, urbanization, and comorbidities related to pneumonia. The variables of age, income level, and urbanization were calculated based on the index date. The definition of comorbidities was diagnosis at least three times outpatient visits a year before the index date. The comorbidities related to pneumonia included diabetes mellitus, hypertension, cerebrovascular disease, arrhythmia, upper respiratory tract infection, heart failure, asthma, COPD, periodontitis, chronic kidney disease, chronic liver disease, alcoholism, Alzheimer's disease, rheumatoid arthritis, cancer, epilepsy, schizophrenia, bipolar disorder, major depressive disorder, and anxiety. The ICD codes of comorbidities were all detailed as Appendix Table 2.

### Statistical Analysis

The SAS software version 9.4 (SAS Institute Inc., Cary, NC, USA) was used for statistical analysis in the study and the statistical significance was defined as the *p* < 0.05. We used descriptive statistics to understand the basic characteristics of subjects, and then the standardized mean difference (SMD) was used to estimate the difference of the distribution between pneumonia patients and comparison. The study investigated the association between antidepressants and pneumonia *via* conditional logistic regression, after being adjusted for the variables, as otherwise the estimation results would have been biased.

## Results

Table 1 is the baseline characteristics of the study subject. After matching, there were a total of 551,975 elderly patients with PD in the study. Among them, 110,395 patients had incident pneumonia and 441,580 patients were without pneumonia, respectively. The age of patients with pneumonia was 80.14 ± 5.85 years old. The characteristics distribution of sex, age, income level, urbanization, and CCI between case group and the comparison were no significant differences with SMD lesser than 0.1 after matching.

**Table 1:** The baseline characteristics of elderly patients with Parkinson's disease after matching.

**Variables**	**Pneumonia**				**Standardized mean difference**
	**Without**		**With**		
	***N***	**%**	***N***	**%**	
Total	441,580	100.00	110,395	100.00	
Sex					0.01
Female	220,311	49.89	54,554	49.42	
Male	221,269	50.11	55,841	50.58	
Age (year) (mean ± SD)	79.94 ± 5.74		80.14 ± 5.85		0.03
65–70	16,185	3.67	4,064	3.68	
70–75	64,198	14.54	16,039	14.53	
75–80	119,984	27.17	30,080	27.25	
80–85	136,585	30.93	34,545	31.29	
85	104,628	23.69	25,667	23.25	
Income level					0
Low income (≤ 21,000)	235,250	53.27	58,886	53.34	
Middle income (21,000–33,000)	95,549	21.64	23,860	21.61	
High income (≥33,000)	110,781	25.09	27,649	25.04	
Urbanization					0
Level 1	100,034	22.65	25,063	22.70	
Level 2	126,121	28.56	31,408	28.45	
Level 3	64,910	14.70	16,344	14.81	
Level 4	80,281	18.18	19,887	18.01	
Level 5	17,518	3.97	4,321	3.91	
Level 6	29,281	6.63	7,462	6.76	
Level 7	23,435	5.31	5,910	5.35	
CCI score					0
0	10,504	2.38	2,629	2.38	
1	45,358	10.27	11,340	10.27	
2	85,582	19.38	21,350	19.34	
≥3	300,136	67.97	75,076	68.01	

Table 2 indicates the association of incident pneumonia and antidepressant use. The incidence rate of pneumonia was 20.14% in current users, 19.59% in recent users, and 21.18% in past users (*p* < 0.001). The adjusted odds ratios (*aOR*) for antidepressants after controlling for sex, age, income level, urbanization, and related comorbidities. Compared with patients not receiving antidepressants, current users receiving antidepressants had a higher risk of incident pneumonia (*aOR* = 1.04, 95% *CI* = 1.02–1.07) and past users (*aOR* = 1.17, 95% *CI* = 1.15–1.19). In terms of TCAs users, compared with patients not receiving TCAs, current users had a lower risk of incident pneumonia (*aOR* = 0.86, 95% *CI* = 0.82–0.90), and recent users (*aOR* = 0.83, 95% *CI* = 0.80–0.87). In terms of MAOIs users, compared with patients not receiving MAOIs, current users had a lower risk of incident pneumonia (*aOR* = 0.88, 95% *CI* = 0.83–0.93), recent users (*aOR* = 0.89, 95% *CI* = 0.85–0.93), and past users had a higher risk of incident pneumonia (*aOR* = 1.09, 95% *CI* = 1.06–1.11). In terms of SSRIs users, current users had a higher risk of incident pneumonia (*aOR* = 1.13, 95% *CI* = 1.01–1.17), recent users (*aOR* = 1.01, 95% *CI* = 1.06–1.13), and past users (*aOR* = 1.19, 95% *CI* = 1.17–1.21), compared with patients not receiving SSRIs. In terms of SNRIs users, past users had a higher risk of incident pneumonia (*aOR* = 1.07, 95% *CI* = 1.03–1.10).

**Table 2:** The incidence rate of pneumonia with antidepressants use.

**Variables**	**Pneumonia**							
	**Without**		**With**		***p* ^b^**	**Multivariate model**		
	***N***	**%**	***N***	**%**		**Adjusted OR**	**95%CI**	***p* ^c^**
**Any types of antidepressants**
No (ref.)	114,684	25.97	25,113	22.75		1		
Current users	48,782	11.05	12,302	22.75	<0.001	1.04	1.02–1.07	<0.001
Recent users	69,505	15.74	16,935	11.14	0.361	1.01	0.99	1.03–0.466
Past users	208,609	47.24	56,045	15.34	<0.001	1.17	1.15–1.19	<0.001
**TCAs** ^**a**^
No (ref.)	314,790	71.29	79,760	72.25		1		
Current users	11,072	2.51	2,307	2.09	<0.001	0.86	0.82–0.90	<0.001
Recent users	16,626	3.77	3,345	3.03	<0.001	0.83	0.80–0.87	<0.001
Past users	99,092	22.44	24,983	22.63	0.176	0.99	0.97–1.01	0.253
**MAOIs** ^**a**^
No (ref.)	386,013	87.42	96,291	87.22		1		
Current users	6,587	1.49	1,398	1.27	<0.001	0.88	0.83–0.93	<0.001
Recent users	9,796	2.22	2,092	1.90	<0.001	0.89	0.85–0.93	<0.001
Past users	39,184	8.87	10,614	9.61	<0.001	1.09	1.06–1.11	<0.001
SSRIs^a^
No (ref.)	321,811	72.88	76,387	69.19		1		
Current users	16,382	3.71	4,517	4.09	<0.001	1.13	1.10–1.17	<0.001
Recent users	23,077	5.23	6,119	5.54	<0.001	1.10	1.06–1.13	<0.001
Past users	80,310	18.19	23,372	21.17	<0.001	1.19	1.17–1.21	<0.001
**SNRIs** ^**a**^
No (ref.)	411,218	93.12	102,346	92.71		1		
Current users	3,877	0.88	920	0.83	0.153	1.01	0.94–1.09	0.832
Recent users	5,631	1.28	1,316	1.19	0.027	1.00	0.94–1.06	0.880
Past users	20,854	4.72	5,813	5.27	<0.001	1.07	1.03–1.10	<0.001

a *TCAs, Tricyclic antidepressants; MAOIs, Monoamine oxidase inhibitors; SSRIs, Selective serotonin reuptake inhibitors; SNRIs, Serotonin norepinephrine reuptake inhibitors*.

b *Chi-square test*.

c *Conditional logistic regression. Extraneous factors adjusted in the model contained sex, age, income level urbanization, and related comorbidities*.

Table 3 indicates that the association of incident pneumonia and individual antidepressant use. In the class of individual TCAs, clomipramine users had a higher risk of incident pneumonia, either current, recent, or past users while amitriptyline had a lower risk of incident pneumonia. In the class of individual MAOIs, selegiline had a lower risk of incident pneumonia, recent users (*aOR* = 0.88, 95% *CI* = 0.83–0.94) and current users (*aOR* = 0.89, 95% *CI* = 0.84–0.94), while past users had a higher risk of incident pneumonia, (*aOR* = 1.10, 95% *CI* = 1.07–1.13). Rasagiline had a lower risk of incident pneumonia, recent users (*aOR* = 0.86, 95% *CI* = 0.76–0.97). In the class of individual SSRIs, fluoxetine, sertraline, and escitalopram users had a higher risk of incident pneumonia, either current, recent, or past users. Paroxetine users had a higher risk of incident pneumonia, current users (*aOR* = 1.11, 95% *CI* = 1.01–1.22). Citalopram users had a higher risk of incident pneumonia, past users (*aOR* = 1.11, 95% *CI* = 1.06–1.15). In the class of individual SNRIs, milnacipran users had a higher risk of incident pneumonia, past users (*aOR* = 1.23, 95% *CI* = 1.05–1.43). Venlafaxine users had a higher risk of incident pneumonia, past users (*aOR* = 1.08, 95% *CI* = 1.04–1.12). Trazodone and mirtazapine users had a higher risk of incident pneumonia, either current, recent, or past users.

**Table 3:** The association of incident pneumonia and individual antidepressants use.

**Variables**	**Pneumonia**							
	**Without**		**With**		***p* ^b^**	**Multivariate model**		
	***N***	**%**	***N***	**%**		**Adjusted OR**	**95% CI**	***p* ^c^**
**TCAs^a^**								
**Amitriptyline**								
No	422,912	95.77	106,234	96.23		1		
Current users	1,308	0.30	263	0.24	0.001	0.87	0.76–1.00	0.051
Recent users	1,900	0.43	377	0.34	<0.001	0.86	0.77–0.96	0.008
Past users	15,460	3.50	3,521	3.19	<0.001	0.90	0.87–0.94	<0.001
**Clomipramine**								
No	440,291	99.71	109,877	99.53		1		
Current users	108	0.02	49	0.04	<0.001	1.53	1.08–2.16	0.016
Recent users	132	0.03	63	0.06	<0.001	1.58	1.16–2.15	0.004
Past users	1,049	0.24	406	0.37	<0.001	1.35	1.20–1.52	<0.001
**Doxepin**								
No	430,025	97.38	107,265	97.16		1		
Current users	830	0.19	212	0.19	0.780	1.05	0.90–1.22	0.561
Recent users	1,192	0.27	313	0.28	0.439	1.07	0.94–1.22	0.292
Past users	9,533	2.16	2,605	2.36	<0.001	1.04	0.99–1.09	0.117
**Imipramine**								
No	335,170	75.90	84,785	76.80		1		
Current users	8,912	2.02	1,804	1.63	<0.001	0.83	0.79–0.88	<0.001
Recent users	13,596	3.08	2,632	2.38	<0.001	0.80	0.77–0.84	<0.001
Past users	83,902	19.00	21,174	19.18	0.174	1.00	0.98–1.01	0.651
**MAOIs^a^**								
**Selegiline**								
No	392,468	88.88	97,662	88.47		1		
Current users	5,440	1.23	1,168	1.06	<0.001	0.88	0.83–0.94	<0.001
Recent users	7,967	1.80	1,721	1.56	<0.001	0.89	0.84–0.94	<0.001
Past users	35,705	8.09	9,844	8.92	<0.001	1.10	1.07–1.13	<0.001
**Rasagiline**								
No	435,447	98.61	109,103	98.83		1		
Current users	1,002	0.23	207	0.19	0.012	0.91	0.78–1.06	0.226
Recent users	1,653	0.37	320	0.29	<0.001	0.86	0.76–0.97	0.011
Past users	3,478	0.79	765	0.69	0.001	0.97	0.89–1.05	0.390
**Moclobemide**								
No	440,638	99.79	110,160	99.79		1		
Current users	149	0.03	25	0.02	0.063	0.75	0.49–1.15	0.184
Recent users	195	0.04	56	0.05	0.360	1.26	0.93–1.71	0.130
Past users	598	0.14	154	0.14	0.743	1.02	0.85–1.22	0.822
**SSRIs^a^**								
**Paroxetine**								
No	421,311	95.41	104,968	95.08		1		
Current users	2,097	0.47	554	0.50	0.247	1.11	1.01–1.22	0.034
Recent users	2,984	0.68	743	0.67	0.921	1.05	0.97–1.14	0.221
Past users	15,188	3.44	4,130	3.74	<0.001	1.00	0.97–1.04	0.868
**Fluoxetine**								
No	410,721	93.01	101,450	91.90		1		
Current users	2,667	0.60	740	0.67	0.012	1.09	1.00–1.19	0.045
Recent users	3,732	0.85	1,022	0.93	0.010	1.09	1.01–1.17	0.023
Past users	24,460	5.54	7,183	6.51	<0.001	1.07	1.04–1.11	<0.001
**Citalopram**								
No	427,542	96.82	106,186	96.19		1		
Current users	1,397	0.32	382	0.35	0.120	1.07	0.95–1.20	0.265
Recent users	1,925	0.44	514	0.47	0.184	1.04	0.94–1.15	0.468
Past users	10,716	2.43	3,313	3.00	<0.001	1.11	1.06–1.15	<0.001
**Fluvoxamine**								
No	435,745	98.68	108,832	98.58		1		
Current users	593	0.13	135	0.12	0.326	0.93	0.77–1.12	0.444
Recent users	798	0.18	178	0.16	0.168	0.91	0.78–1.08	0.289
Past users	4,444	1.01	1,250	1.13	<0.001	1.00	0.94–1.07	0.917
**Sertraline**								
No	397,545	90.03	97,044	87.91		1		
Current users	5,062	1.15	1,555	1.41	<0.001	1.26	1.18–1.33	<0.001
Recent users	7,380	1.67	2,064	1.87	<0.001	1.15	1.10–1.21	<0.001
Past users	31,593	7.15	9,732	8.82	<0.001	1.20	1.17–1.23	<0.001
**Escitalopram**								
No	407,346	92.25	101,209	91.68		1		
Current users	4,790	1.08	1,230	1.11	0.400	1.09	1.021.16	0.012
Recent users	6,846	1.55	1,737	1.57	0.579	1.07	1.02–1.13	0.013
Past users	22,598	5.12	6,219	5.63	<0.001	1.07	1.04–1.11	<0.001
**SNRIs^a^**								
**Duloxetine**								
No	427,180	96.74	106,904	96.84		1		
Current users	1,895	0.43	430	0.39	0.069	1.00	0.90–1.12	0.967
Recent users	2,767	0.63	585	0.53	<0.001	0.94	0.86–1.03	0.165
Past users	9,738	2.21	2,476	2.24	0.448	1.02	0.97–1.07	0.470
**Milnacipran**								
No	440,751	99.81	110,106	99.74		1		
Current users	65	0.01	23	0.02	0.150	1.54	0.95–2.51	0.081
Recent users	90	0.02	31	0.03	0.122	1.42	0.93–2.15	0.102
Past users	674	0.15	235	0.21	<0.001	1.23	1.05–1.43	0.010
**Venlafaxine**								
No	424,831	96.21	105,665	95.72		1		
Current users	1,931	0.44	473	0.43	0.690	1.00	0.91–1.11	0.956
Recent users	2,815	0.64	703	0.64	0.980	1.03	0.94–1.12	0.574
Past users	12,003	2.72	3,554	3.22	<0.001	1.08	1.04–1.12	<0.001
**Other antidepressants**								
**Trazodone**								
No	345,595	78.26	83,684	75.80		1		
Current users	11,096	2.51	3,109	2.82	<0.001	1.13	1.09–1.18	<0.001
Recent users	15,950	3.61	4,292	3.89	<0.001	1.10	1.06–1.14	<0.001
Past users	68,939	15.61	19,310	17.49	<0.001	1.09	1.07–1.12	<0.001
**Mirtazapine**								
No	417,178	94.47	103,312	93.58		1		
Current users	3,069	0.70	916	0.83	<0.001	1.27	1.18–1.37	<0.001
Recent users	4,490	1.02	1,240	1.12	0.002	1.19	1.11–1.27	<0.001
Past users	16,843	3.81	4,927	4.46	<0.001	1.12	1.08–1.16	<0.001

a *TCAs, Tricyclic antidepressants; MAOIs, Monoamine oxidase inhibitors; SSRIs, Selective serotonin reuptake inhibitors; SNRIs, Serotonin norepinephrine reuptake inhibitors*.

b *Chi-square test*.

c *Conditional logistic regression. Extraneous factors adjusted in the model contained sex, age, income level urbanization, and related comorbidities*.

## Discussion

Various drug properties have different pharmaceutical effects that can substantially affect biological activities. These individual antidepressants may have different action mechanisms that drive the development of pneumonia. This case-control study analyzed the data of 551,975 older adult patients with PD between 2001 and 2018 in Taiwan. Some risk factors for pneumonia (e.g., COPD) had not enrolled as matching variables in the study. The study used the statistical method to adjust the pneumonia-related comorbidities to estimate the correlation between antidepressants and pneumonia, instead of enrolling as matching variables. In our study, we found that the risk of pneumonia was associated with the use of individuals belonging to different classes of antidepressants among older patients with PD after controlling all related variables (sex, age, income level, urbanization, and related comorbidities). The users of TCAs (such as, amitriptyline and imipramine) had a lower risk of incident pneumonia, whereas the users of clomipramine had a higher risk of incident pneumonia. Recent use of MAOIs (such as, selegiline and rasagiline) could reduce the risk of pneumonia. The users of SSRIs (such as, fluoxetine, sertraline, escitalopram, paroxetine, and citalopram) had a higher risk of incident pneumonia. In addition, the use of SNRIs (such as, milnacipran and venlafaxine) could increase the risk of pneumonia. Other antidepressants (such as, trazodone and mirtazapine) also increased the risk of pneumonia.

Oropharyngeal dysphagia is associated with a higher risk of pneumonia in the patients of PD (19). Depression was noted in ~30–40% of patients with PD, and these patients may receive treatment for depression, such as antidepressants (20). A meta-analysis indicated a significant increase in pro-inflammatory cytokines, such as tumor necrosis factor (TNF)-α and interleukin-6 (IL-6), in patients with depression (21). Alterations in the *Th1/Th2 balance* might result from maladjustment in depression (22), whereas antidepressants may act as an immunomodulator (8). The immunomodulatory effects of antidepressants on the Th1/Th2 balance require further systematic evaluation. Patients with PD who develop depression receive antidepressants.

There are several studies that have shown inconsistent findings on the correlation between antidepressant use and pneumonia (11, 23, 24). A meta-analysis study showed that treatment with antidepressants was associated with a decreased level of TNF-α, but only in those who responded to the treatment (25). This study indicated that inflammatory markers improve only in patients whose depression symptom improves after antidepressant treatment. In general, antidepressant medications tend to reduce the levels of pro-inflammatory factors, such as TNF-α, IL-1β, and IL-6, found in many patients with depressive disorders (8). Another study showed that the odds ratio (*OR*) for any antidepressant use was 1.61 (95% *CI* = 1.46–1.78). After further adjustment for comorbidities, the OR was 0.89 (95% *CI* = 0.79–1.00). This study indicated that the use of antidepressants among older patients increases the risk of hospitalization for pneumonia (23). The result may be biased because this study did not investigate whether the risk of pneumonia is associated with different types of antidepressants. Investigating the risk of pneumonia resulting from the use of various types of antidepressants is necessary.

Our study results revealed that the current users of TCAs had a lower risk of incident pneumonia. Some studies have shown that TCAs can inhibit the secretion of Th1-type cytokines (e.g., IL-1β, IL-2, TNF-α, and IFN-γ) and stimulate the production of Th2-type cytokines (e.g., IL-10) (22). Our results indicated that the use of TCAs, such as amitriptyline and imipramine can reduce the risk of pneumonia in patients with PD. This phenomenon is supported by the finding of a previous study that indicated that TCAs may display anti-inflammatory effects on the TNF-α system (7). Because imipramine attenuates neuro-inflammatory signaling and reverses stress-induced social avoidance (26), it may exert its effect, in part, by down-regulating microglial activation (26). By contrast, our results indicated that the current, recent, and past users of clomipramine had an increased risk of pneumonia. This phenomenon may be associated with the following finding of a population-based case-cohort study conducted in the Netherlands: clomipramine was associated with a higher risk of agranulocytosis (*OR* = 20.0, 95% *CI* = 6.1–57.6) (27). However, the real mechanism remains unknown and should be further investigated.

The current and recent users of MAOIs had a lower risk of incident pneumonia, whereas the past users of MAOIs had a higher risk of incident pneumonia. Previous studies have indicated that monoamine oxidase (MAO) is involved in neuroinflammation (28). Selegiline and rasagiline are MAO-B inhibitors that reduce the breakdown of dopamine in the brain (29). MAO-B inhibition appears to mainly enhance the dopaminergic system (22). Our study results indicated that the use of selegiline could reduce the risk of pneumonia in patients with PD during current use and recent use. Moreover, rasagiline could reduce the risk of pneumonia in patients with PD during recent use. This phenomenon is supported by the findings of previous studies that have reported that selegiline could reduce the production of TNF-α and stimulate the biosynthesis of IL-6 and IL-1β (22), and rasagiline exerts an antiapoptotic effect (30). Dopamine deficiency in the brain is a major cause of deregulation of motor symptoms in patients with PD (31). Both selegiline and rasagiline demonstrate the neuroprotective activity that may slow the progression of PD (32, 33). It is conflicting from the clinical data that long-term use of selegiline (34–36). A study indicated that the early combination of selegiline and levodopa proved to be clearly superior to levodopa monotherapy, while long-term side effects appeared occurred in the selegiline group, although the difference was not significant (35). A United Kingdom research showed that mortality was significantly higher in the selegiline and levodopa combination treatment, casting doubts on its chronic use in PD (36). More large-scale prospective studies are warranted to further clarify an understanding of the long-term use safety of selegiline.

Our study results indicated that the current, recent, and past users of SSRIs had a higher risk of incident pneumonia. SSRIs can inhibit serotonin reuptake and may enhance serotonin activity, which in turn exerts immunostimulatory effects on Th1 cytokines and concomitant immunoinhibitory effects on Th2 cytokines (37). SSRIs exert weak anticholinergic effects (9). However, our study findings revealed that fluoxetine, sertraline, and escitalopram could increase the risk of pneumonia in patients with PD during current use, recent use, and past use. Moreover, paroxetine could increase the risk of pneumonia during current use, and citalopram could increase the risk of pneumonia during past use. A higher risk of pneumonia may be associated with the finding of previous studies that SSRIs, such as sertraline, paroxetine, and citalopram may exert immunosuppressive effects and increase the risk of infections (38, 39). Subchronic pretreatment with citalopram reduces mood symptoms induced by acute immune system activation with endotoxins without inhibiting the peripheral immune response (40). A case report showed that agranulocytosis was caused by fluoxetine (41). However, the actual underlying mechanism remains unclear and should be investigated in the future.

In our study, we found that past users of SNRIs had a higher risk of incident pneumonia. The finding can be attributed to the anti-inflammatory action of SNRIs (42). A meta-analysis reported that a noradrenalin reuptake inhibitor may suppress Th1-type cytokines and shift the balance toward humoral immunity (21). SNRIs exert a weak anticholinergic effect (9). However, our study results indicated that milnacipran and venlafaxine could increase the risk of pneumonia among patients with PD during past use. This finding may be attributed to the complex effect of venlafaxine on cytokine levels. Venlafaxine acts as a real SNRI at a higher dose, but it mainly blocks the reuptake of serotonin at a lower dose; at a high dose, venlafaxine blocks the reuptake of serotonin and NE to the same extent (43). Epinephrine exposure may alter the inflammatory response, potentially contributing to adverse clinical outcomes (44). A case report indicated that venlafaxine induced acute eosinophilic pneumonia (45).

Trazodone has been classified as a serotonin antagonist/reuptake inhibitor based on its antagonism of 5HT2A, 5HT2C, and serotonin reuptake receptors. Trazodone has hypnotic actions at low doses due to the blockade of 5-HT2A receptors as well as H1 histamine receptors and α1 adrenergic receptors (46) and is currently the most commonly prescribed antidepressant for primary insomnia (47). Our study result revealed that the use of trazodone increased the risk of pneumonia in patients with PD. The risk of pneumonia is associated with the hypnotic actions of trazodone. Trazodone at a high dose appeared to reduce the levels of TNF-α, IL-1β, and IFN-γ (22). Mirtazapine is a tetracyclic antidepressant, acts as a potent inhibitor of 5HT2 and 5HT3, and is a central α2-adrenergic and histamine H1 receptor. The action of mirtazapine on 5HT2 and H1 might contribute to its high sedating activity (22). Our study results indicated that the use of mirtazapine increased the risk of pneumonia in patients with PD. This phenomenon is in agreement with that of a previous study that indicated that mirtazapine increased the inflammatory markers such as IL-1β, IL-6, and TNF-α, inhibited IFN-γ production, and increased IL-4 production (48).

To the best of our knowledge, this is the first study to identify individual antidepressants, as risk factors for pneumonia in patients with PD subjects. This study has several strengths. First, we included the entire Taiwanese population in this study; thus, the large sample size is more representative of the population and can provide high-quality data to study this issue. This study is a nationwide population-based case-control study with nearly complete follow-up information with regard to healthcare institutes for the whole study population; the dataset used in this study is routinely monitored for diagnostic accuracy by the NHI Bureau of Taiwan. Second, the follow-up period of this study was divided into current use, recent use, and past use to investigate the relationship between the risk of pneumonia and antidepressant use in older patients with PD. This categorization helped in understanding the relationship between the risk of pneumonia during current, recent, and past use. Third, this study investigated whether the risk of pneumonia is associated with individual antidepressants and various types of antidepressants. Thus, we could understand the relationship between the risk of pneumonia and individual and different classes of antidepressants. Fourth, we investigated whether comorbidities are risk factors for pneumonia.

This study also has some limitations that should be addressed. First, some factors affecting pneumonia could not be obtained from the LHID, such as alcohol consumption behavior, smoking behavior, chest X-ray results, etiology of pneumonia, and laboratory parameters finding. The LHID only can present health insurance declaration information, and self-pay medical information cannot be obtained. Thus, the status of antidepressant use may be underestimated. The risk of pneumonia among elderly patients with PD receiving antidepressants is possible with the poorer physical condition, rather than antidepressants. Therefore, this study which is an epidemiology observational study based on secondary data analysis reduced the confounding and study bias by statistical matching methods and adjusting comorbidity disease. In addition, the severity of PD and the disease duration of PD may also affect it. This study was a nationwide population-based study. Thus, the study results still have accuracy and representativeness, although there were a number of potential biases that cannot be included in the analysis. Third, the inclusion of the prevalence of antidepressant users could have potentially underestimated the overall risk because they might have a developed tolerance for pneumonia. Fourth, the study only included ICD codes to define diseases without any medical procedure codes. This might have resulted in over-diagnosis. Finally, because this is an observational study, the cause-and-effect relationship between antidepressant usage and pneumonia could not be determined. Future studies should obtain more information from other relational databases or questionnaires to analyze the cause-and-effect relationship.

## Conclusion

The incident pneumonia is associated with the use of individuals of different classes of antidepressants. The use of TCAs (such as amitriptyline and imipramine) had a lower odds of incident pneumonia. The use of MAOIs (such as selegiline and rasagiline) had a lower odds of pneumonia during recent use. The use of SSRIs (such as fluoxetine, sertraline, escitalopram, paroxetine, and citalopram) and SNRIs (such as milnacipran and venlafaxine) had a higher odds of incident pneumonia.

## Data Availability Statement

The data analyzed in this study is subject to the following licenses/restrictions: The National Health Insurance Database used to support the findings of this study was provided by the Health and Welfare Data Science Center, Ministry of Health and Welfare (HWDC, MOHW) under license and so cannot be made freely available. Requests for access to these data should be made to HWDC. Requests to access these datasets should be directed to https://dep.mohw.gov.tw/dos/np-2497-113.html.

## Author Contributions

Conceptualization was performed by K-HH, W-YK, and C-YL. Data curation was performed by Y-HK. Formal analysis was performed by Y-CC and T-HT. Funding acquisition was done by K-HH and C-YL. Investigation was done by W-YK. Methodology was done by K-HH, W-YK, Y-HK, Y-CC, and C-YL. Validation was done by Y-HK, Y-CC, and C-YL. Writing—original draft was done by K-HH, W-YK, and C-YL. Writing—review and editing was done by C-YL. All authors contributed to the article and approved the submitted version.

## Funding

This research was funded by the Ministry of Science and Technology Taiwan (MOST 109-2410-H-039-004; MOST 110-2410-H-040-002), Chung Shan Medical University Taiwan (CSMU-INT-109-07), and China Medical University Taiwan (CMU108-ASIA-13; CMU109-MF-120).

## Conflict of Interest

The authors declare that the research was conducted in the absence of any commercial or financial relationships that could be construed as a potential conflict of interest.

## Publisher's Note

All claims expressed in this article are solely those of the authors and do not necessarily represent those of their affiliated organizations, or those of the publisher, the editors and the reviewers. Any product that may be evaluated in this article, or claim that may be made by its manufacturer, is not guaranteed or endorsed by the publisher.
