# Supplementary material for: Antidepressants Usage and Risk of Pneumonia Among Elderly Patients With the Parkinson's Disease: A Population-Based Case-Control Study
Source: Front Med (Lausanne). 2022 Feb 18;9:740182. doi: 10.3389/fmed.2022.740182 (PMC8896435; doi:10.3389/fmed.2022.740182)
Supplement: Supplementary file 1 [file Table_1.pdf]

Appendix table 1. The Anatomic Therapeutic Chemical classification (ATC) system codes for antidepressants.

Antidepressants	ATC codes
Tricyclic antidepressants (TCAs)	
Amitriptyline	N06AA09
Clomipramine	N06AA04
Doxepin	N06AA12, D04AX01
Imipramine	N06AA02
Monoamine oxidase inhibitors (MAOIs)	
Isocarboxazid	N06AF01
Selegiline	N04BD01
Rasagiline	N04BD02
Tranylcypromine	N06AF04
Moclobemide	N06AG02
Selective serotonin reuptake inhibitors (SSRIs)	
Paroxetine	N06AB05
Fluoxetine	N06AB03
Citalopram	N06AB04
Fluvoxamine	N06AB08
Sertraline	N06AB06
Escitalopram	N06AB10
Serotonin norepinephrine reuptake inhibitors (SNRIs)	
Duloxetine	N06AX21
Milnacipran	N06AX17
Venlafaxine	N06AX16
Other antidepressants	
Trazodone	N06AX05
Mirtazapine	N06AX11
