# Supplementary material for: Antidepressants Usage and Risk of Pneumonia Among Elderly Patients With the Parkinson's Disease: A Population-Based Case-Control Study
Source: Front Med (Lausanne). 2022 Feb 18;9:740182. doi: 10.3389/fmed.2022.740182 (PMC8896435; doi:10.3389/fmed.2022.740182)
Supplement: Supplementary file 2 [file Table_2.pdf]

Appendix table 2. The International Classification of Diseases (ICD) codes for comorbidities.

Comorbidities	ICD codes
Diabetes mellitus	ICD-9-CM: 250<br>ICD-10-CM: E08-E13
Hypertension	ICD-9-CM: 401-405<br>ICD-10-CM: I10-I13, I15
Cerebrovascular disease	ICD-9 CM: 430-438<br>ICD-10-CM: I60-I69
Arrhythmia	ICD-9-CM: 427<br>ICD-10-CM: I47-I49
Upper respiratory tract infection	ICD-9-CM: 465.9<br>ICD-10-CM: J00-06, J30-39
Heart failure	ICD-9-CM: 428.0<br>ICD-10-CM: I50
Asthma	ICD-9-CM: 493<br>ICD-10-CM: J45
Chronic obstructive pulmonary disease	ICD-9-CM: 490-492, 494-496<br>ICD-10-CM: J40-J44, J47
Periodontitis	ICD-9-CM: 523<br>ICD-10-CM: K05.4
Chronic kidney disease	ICD-9-CM: 585<br>ICD-10-CM: N18
Chronic liver disease	ICD-9-CM: 571<br>ICD-10-CM: K70-K76
Alcoholism	ICD-9-CM: 303<br>ICD-10-CM: F10.2
Alzheimer’s disease	ICD-9-CM: 331.0, 290.1<br>ICD-10-CM: G30, F00
Rheumatoid arthritis	ICD-9-CM: 714<br>ICD-10-CM: M05-M06, M45
Cancer	ICD-9-CM: 140-239<br>ICD-10-CM: C00-C97
Epilepsy	ICD-9-CM: 345<br>ICD-10-CM: G40-G41
Schizophrenia	ICD-9-CM: 295-295.65, 295.8-295.95<br>ICD-10-CM: F20-F20.9
Bipolar disorder	ICD-9-CM: 296.7<br>ICD-10-CM: F31.9
Major depressive disorder	ICD-9-CM: 296.3<br>ICD-10-CM: F32.9
Anxiety	ICD-9-CM: 300.0<br>ICD-10-CM: F40, F41
